# Paediatric bone marrow mesenchymal stem cells support acute myeloid leukaemia cell survival and enhance chemoresistance via contact‐independent mechanism

**DOI:** 10.1111/bjh.19884

**Published:** 2024-11-10

**Authors:** Alison Laing, Ahmed Elmarghany, Arwa A. Alghaith, Aya Gouma, Thomas Stevens, Alexander Winton, Jennifer Cassels, Cassie J. Clarke, Claire Schwab, Christine J. Harrison, Brenda Gibson, Karen Keeshan

**Affiliations:** ^1^ Paul O'Gorman Leukaemia Research Centre, School of Cancer Sciences University of Glasgow Glasgow UK; ^2^ Wolfson Wohl Cancer Research Centre, School of Cancer Sciences University of Glasgow Glasgow UK; ^3^ Haematology Department Queen Elizabeth University Hospital Glasgow UK; ^4^ Clinical Pathology Department Mansoura University Mansoura Egypt; ^5^ Clinical Pathology Department Zagazig University Zagazig Egypt; ^6^ Cancer Research UK Scotland Institute Garscube Estate Glasgow UK; ^7^ Leukaemia Research Cytogenetics Group, Translational and Clinical Research Institute Newcastle University Centre for Cancer Newcastle Upon Tyne UK; ^8^ Royal Hospital for Children Glasgow UK

**Keywords:** AML, chemosensitivity, childhood leukaemia, mesenchymal cells

## Abstract

Children diagnosed with acute myeloid leukaemia (paediatric AML [pAML]) have limited treatment options and relapse rates due to chemoresistance and refractory disease are over 30%. Current treatment is cytotoxic and in itself has long‐lasting harsh side effects. New, less toxic treatments are needed. The bone marrow microenvironment provides chemoprotection to leukaemic cells through cell communication and interaction with mesenchymal stem cells (MSCs), but this is not well defined in pAML. Using primary patient material, we identify a cell contact‐independent mechanism of MSC‐mediated chemoprotection involving extrinsic soluble factors that is abrogated through inhibition of the JAK/STAT and ERK pathways.

Recent experimental data point towards an age‐dependent remodelling of the bone marrow (BM) niche during leukaemogenesis^1^; therefore, it is important to understand the unique biology of the niche in childhood acute myeloid leukaemia (AML). The BM niche finely regulates haematopoiesis through a balance of self‐renewal and differentiation and is controlled via signals from the surrounding BM microenvironment (BMM) comprising the extracellular matrix (ECM), neighbouring cells, cytokines, hormones and mechanical forces. Under both healthy and leukaemic states, cells communicate with their BMM through the secretion of cytokines and other soluble factors, extracellular vesicles and by direct cellular contact.^2^, ^3^ Adult mesenchymal stem cells (aMSCs) in the BM have been shown to be crucial for healthy haematopoiesis by providing physical support and secreting soluble factors. It has been well studied that the adult BMM is crucial for supporting AML progression^4^ and the signals that support normal haematopoiesis are remodelled by AML cells to create a pro‐leukaemic BM niche.^5^, ^6^ Indeed, aMSCs provide therapy resistance in adult AML through inflammatory cytokines and immune evasion including protection from natural killer (NK) cell‐mediated killing, and escape from anti‐leukaemic effector lymphocytes.^5^ However, the neonatal BMM is less defined and its role in paediatric AML is largely unknown. Paediatric MSCs (pMSCs) create physical connections with paediatric AML (pAML) cells and result in the transcriptional reprogramming of the pMSCs,^7^ but how pMSCs and the surrounding BMM alter pAML cells is not fully understood.

We isolated pMSCs from BM pAML mononuclear cells using the plastic adherence method and characterized them as having a CD45−Lin−CD90+CD105+CD73+ phenotype and ability to differentiate into three cell lineages: adipocytes, osteoblasts and chondrocytes, as per the guidelines of the internal Society for Cellular Therapy^8^ (Data S1; Figure S1). In some paediatric leukaemias, it was reported that the pMSCs expressed the leukaemic oncofusion [*KMT2A::AFF1* B‐ALL, the t(4;11)(q21;q23) translocation was detected] suggesting non‐haematopoietic expression may influence the BMM.^9^ However, in line with other pAML research,^7^ we did not detect any of the cytogenetic abnormalities similar to the AML blast identifiers in the pMSCs. To investigate the relationship of pAML cells and pMSCs, a primary 2D coculture (CC) was created with cells in direct contact with and without cytokine supplementation. The pMSCs significantly increased the survival of CD45+ pAML blasts compared to liquid culture (LC) (Figure 1A–C). This effect on pAML cells was observed in both autologous and allogenic pMSC CC settings. Previous work has shown that the BMM and MSCs provide chemoprotection to AML cells.^10^ We show here that the pMSCs increased the survival of the paediatric Kasumi‐1 AML cell line following treatment with the standard chemotherapeutic drugs cytarabine (Ara‐C) and the anti‐CD33 antibody drug conjugate gemtuzumab ozogamicin (GO). Ara‐C is a standard of care cytotoxic drug and GO is now in front‐line AML combination therapy and its dosing schedule has recently been assessed in children with AML within the Myechild01 clinical trial (NCT02724163). Following either Ara‐C or GO treatment, pAML cells in CC were less apoptotic as measured by Annexin−DAPI− and by cell cycle with less cells in sub‐G1 phase and more cells in G1‐S‐G2/M cycle. The chemoprotection was evident from pMSCs from different patients as well as from the stromal cell line HS5 (Figure 1D,E; Figure S2). The surviving CD45+ AML cells had increased clonogenic activity, where following washout of the drug and plating in a colony‐forming cell assay, the colonies from cells in CC with and without drug were much larger and were significantly increased in number (Figure S2). To understand the biological changes in the pAML cells due to pMSC interactions, we performed transcriptional profiling by RNA‐sequencing of six patient pAML blast samples following CC with pMSCs compared to LC. Differential gene expression and gene set enrichment analysis revealed significant enrichment of several genes and pathways relating to the ECM, cell adhesion, integrin‐binding, cytokine regulation and secretion (Figure 1F,G). Cytokines are key components of the BMM that induce the activation of several common intracellular signalling pathways that promote cell survival, including the JAK/STAT, PI3K and ERK1/2 pathways. These were significantly enriched signalling pathways in the AML blasts from CC. Leading‐edge analysis of the ERK1/2‐enriched pathways showed that several genes that are highly expressed are related to the ECM, including *FN1*, *ITGAV*, *CCN1* and *CCN2* (Figure S3). These data suggest that the pMSCs may alter the cytokine/chemokine secretome and the ECM to alter the BMM leukaemic niche.

**FIGURE 1:**
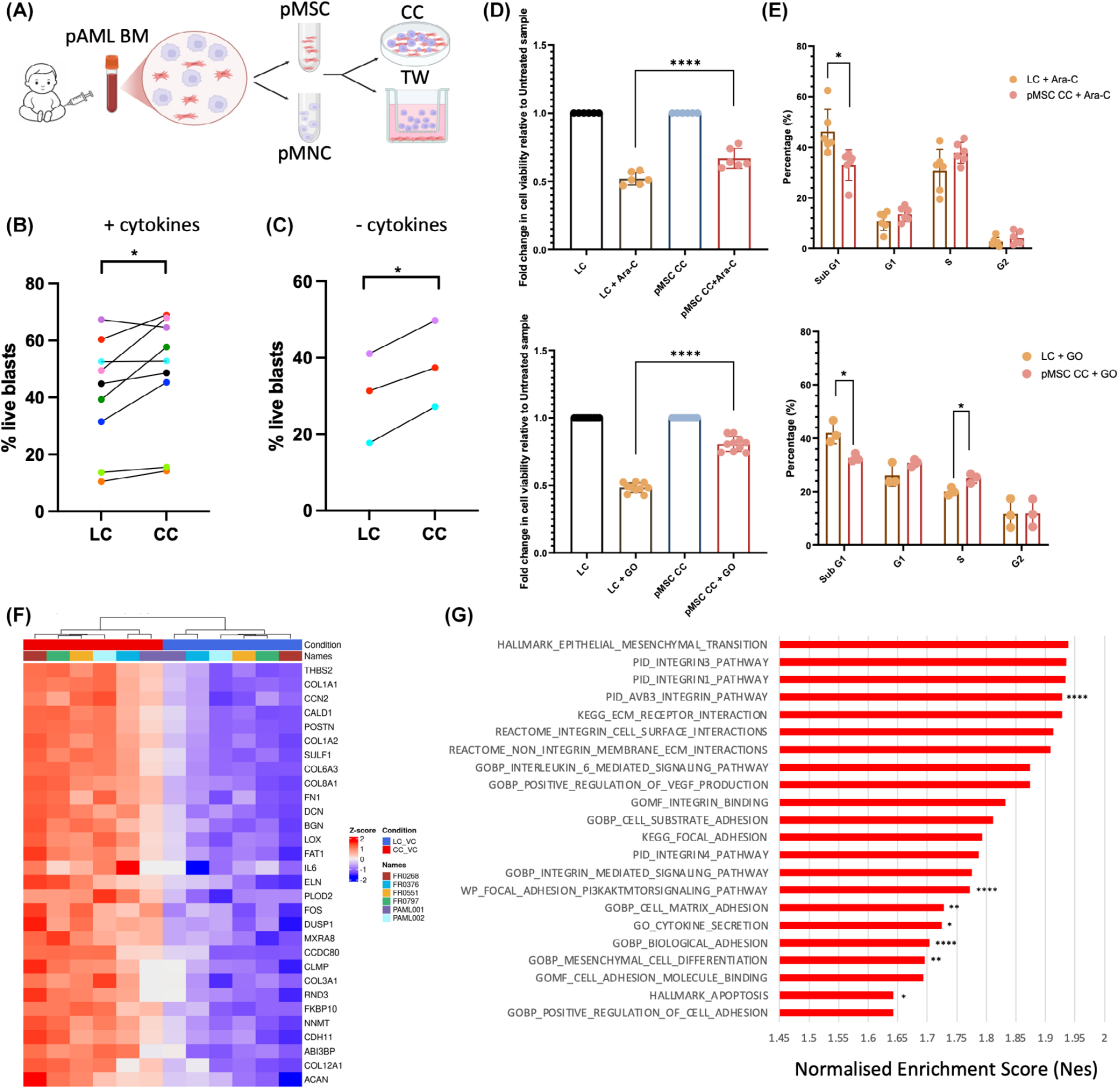
(A) Schematic of primary pAML co‐culture system illustrating that primary AML BM samples were used to isolate pMSC and mononuclear cells (MNC). Primary pMSC from passage 2 to 5 were seeded at a density of 10^4^/cm^2^ in pMSC media and left for 72 h until >90% confluent. pMSC media were then removed and MNC were added in human culture media (HCM), with and without additional cytokines. (B, C) Combined data from primary AML samples (*n* = 9) comparing the percentage of live CD45+ blasts from apoptosis assay analysis in LC and CC with additional cytokine supplementation (B) and without additional cytokine supplementation. (C) Each patient sample has been allocated a different coloured dot in the graphs for easier identification of sample pairs. *p*‐Values were determined using a two‐sided paired Student's *t*‐test (*p < 0.05). (D) Graphs show fold change in the percentage of live (Annexin V−/DAPI−) Kasumi‐1 AML cells from an apoptosis assay and (E) graph shows percentage of cells in each phase of the cell cycle for Kasumi‐1, in LC and pMSC CC after Ara‐C drug treatment (top panels), and in LC and pMSC CC after GO drug treatment (bottom panels) compared to the respective untreated sample. *n* = 3 biological replicates. For LC and pMSC CC after GO drug treatment, *N* = 10 including PAML001, PAML002 and PAML028 pMSC. *p*‐Values were determined using a two‐sided unpaired Student's *t*‐test (*p < 0.05, **p < 0.01, ****p < 0.0001). (F) Heatmap of RNA sequencing data showing the top 30 significantly differentially expressed genes (lowest *p*.adj value) for primary P‐AML CD45‐positive blasts (*n* = 6) in pMSC CC compared to LC. *Z*‐scores are the normalized transformed gene counts for each sample in the different culture conditions. (G) Bar chart showing the normalized enrichment score (NES) for statistically significant biological pathways identified by gene set enrichment using fGSEA (*p* adjusted value of <0.05, *p*.adj * <0.05, ** <0.01, **** < 0.0001). AML, acute myeloid leukaemia; Ara‐C, cytarabine; BM, bone marrow; CC, coculture; GO, gemtuzumab ozogamicin; LC, liquid culture; pMSC, paediatric mesenchymal stem cell. [Colour figure can be viewed at wileyonlinelibrary.com]

To address whether pMSCs alter the cytokine/chemokine secretome, we performed an unbiased multiplex immunoassay (45‐plex and validation 8‐plex immunoassays) using a total of nine different (allogenic and autologous) primary pAMLs and pMSCs following CC for 48 and 72 h. From both these screens, there were four cytokines that had higher levels within the CC compared to LC of pAMLs or pMSCs alone: interleukin 6 (IL‐6), hepatocyte growth factor, stromal cell‐derived factor 1 (SDF‐1) and vascular endothelial growth factor A (Figure 2A; Figure S4). One of the most widely studied chemokine signalling pathways in the BMM is CXCL12 (SDF‐1)/CXCR4, which under normal conditions contributes to the maintenance of quiescent haematopoietic stem cells but has been linked to the promotion of AML cell proliferation and survival under disease conditions.^3^, ^5^ Additionally, MSCs secrete cytokine IL‐6 in the leukaemic BMM, which has been associated with the activation of pro‐survival signalling pathways and increased chemoprotection.^11^, ^12^ In clinical settings, IL‐6 plasma levels were shown to be significantly higher at relapse in pAML patients in a 5‐year event‐free survival analysis.^13^ Together the secretome and transcriptomic data suggest an altered secretome/ECM in the leukaemic niche due to pMSC and pAML cellular interaction.

**FIGURE 2:**
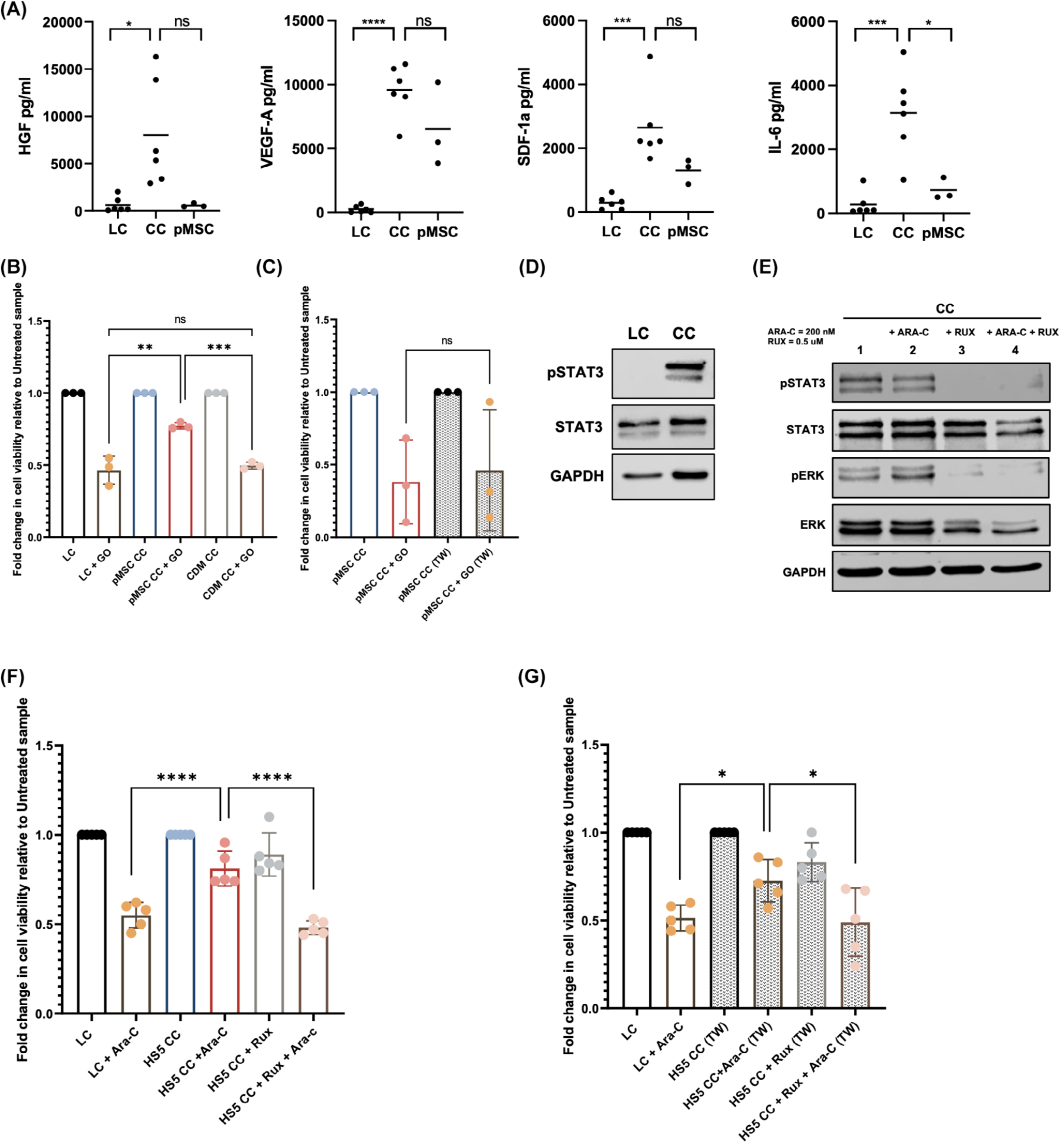
(A) Graph showing four significantly different cytokine concentrations of IL‐6, HGF, VEGF‐A and SDF‐1a at 72 h for six pAML samples and three pMSC samples. *p*‐Values were determined using a two‐sided unpaired Student's *t*‐test (*p < 0.05, ***p < 0.001, ****p < 0.0001). (B) Fold change in percentage of live (Annexin V−/DAPI−) Kasumi‐1 AML cells from an apoptosis assay in LC, pMSC CC and CDM CC after GO drug treatment compared to the respective untreated sample (*n* = 3 biological replicates). *p*‐Values were determined using a two‐sided unpaired Student's *t*‐test (** <0.01, *** <0.001). (C) Fold change in percentage of live (Annexin V−/DAPI−) CD45‐positive primary paediatric AML blasts in pMSC CC and pMSC transwell (TW) CC following GO drug treatment compared to the respective untreated sample for three primary samples (*n* = 3). *p*‐Values were determined using a two‐sided paired Student's *t*‐test. (D) Western blot images of phosphorylated STAT3 and total STAT3 levels in Kasumi‐1 cells lysates from LC and CC. GAPDH as a protein loading control. (E) Western blot images from Kasumi‐1 AML cell lysates from cell treated with the indicated concentration of Ara‐C and RUX in CC. (F) Fold change in percentage of live (Annexin V−/DAPI−) Kasumi‐1 AML cells in LC and HS5 CC following Ara‐C treatment with or without RUX treatment compared to the respective untreated sample (*n* = 5 biological replicates) (****p < 0.0001). (G) Fold change in percentage of live (Annexin V−/DAPI−) Kasumi‐1 AML cells in LC and HS5 transwell (TW) CC following Ara‐C treatment with or without RUX treatment (*n* = 5 biological replicates). *p*‐Values were determined using a two‐sided unpaired Student's *t*‐test (*p < 0.05). AML, acute myeloid leukaemia; Ara‐C, cytarabine; CC, coculture; CDM, cell‐derived matrices; GO, gemtuzumab ozogamicin; HGF, hepatocyte growth factor; IL‐6, interleukin 6; LC, liquid culture; pMSC, paediatric mesenchymal stem cell; SDF, stromal cell‐derived factor 1; VEGF, vascular endothelial growth factor A. [Colour figure can be viewed at wileyonlinelibrary.com]

Whether the chemoprotection is cell contact dependent or independent (through the ECM or secreted/soluble factors) is important to understand the mechanism involved. To test this, we generated cell‐derived matrices (CDM) from pMSCs alone to reconstitute the ECM niche in vitro from pMSCs, with fibronectin and collagen staining (FN1 and COL1A) validating successful creation of the CDM (Figure S4D). Following GO treatment, the CDM derived from the pMSCs alone was unable to provide the same chemoprotection to pAML Kasumi‐1 cells as compared to pMSCs in direct contact (Figure 2B). Furthermore, the chemoprotection afforded to pAML cells in CC was similar whether cells were allowed in direct contact or in the absence of contact using a transwell in CC (Figure 2C). These data support that the pMSCs secretome provides chemoprotection that is cell contact independent.

Adult mesenchymal stem cells in CC with adult AML cell lines were recently shown to induce AML chemoresistance and epithelial–mesenchymal transition‐like programme through IL‐6 signalling.^11^ Many cytokines, growth factors and their receptors can activate anti‐apoptotic and pro‐survival signalling pathways, including the JAK/STAT and ERK1/2 pathways, which can alter the molecular and biological function of AML cells. In adult AML BM, increased inflammatory cytokine production (e.g. IL‐6, IL‐8, tumour necrosis factor α) led to JAK/STAT pathway activation, but monotherapy with a JAK/STAT inhibitor lacked substantial antileukaemic activity in vivo.^14^ We show that following CC with pMSC, activation of the JAK/STAT signalling pathway is observed (Figure 2D). To test whether the pMSCs are mechanistically leading to JAK/STAT pathway‐mediated chemoprotection, we treated the CC with the chemotherapeutic drug and the JAK/STAT pathway inhibitor, ruxolitinib (RUX) that also inhibits ERK1/2 pathway.^15^ RUX treatment in CC efficiently blocked the activation of the JAK/STAT and ERK pathways as shown by a block in phosphorylated STAT3 and ERK1/2 (Figure 2E). Following Ara‐C and RUX treatment, the pMSCs were no longer able to provide the same chemoprotection to the pAML cells (Figure 2F). Furthermore, contact‐independent chemoprotection was abrogated with RUX as demonstrated by Ara‐C and RUX combination treatment in CC in the presence of the transwell (Figure 2G). These data show that combination treatment was able to negate the chemoprotective effects the pMSCs provide to pAML cells in the absence of direct cell contact.

In summary, the data reveal a highly supportive and chemoprotective role for pMSCs in pAML, through alteration of pAML cells' molecular profile and contact‐independent‐driven therapy resistance involving soluble/secreted factors.

## AUTHOR CONTRIBUTIONS

AL and KK conceived and designed the experiments. AL, AE, AAA, AG, TS, AW, JC, CJC and CS performed the experiments, analysed and curated the data. BG and CJH provided resources. KK wrote the manuscript. AL, KK and BG secured the funding. All authors reviewed the manuscript. Authorship abides by the CRediT.

## CONFLICT OF INTEREST STATEMENT

All authors declare no conflict of interest.

## Supporting information


Data S1.

## Data Availability

The RNA‐seq data discussed in this publication have been deposited in the GEO databases under the accession code GSE277382.
