# Supplementary material for: Paediatric bone marrow mesenchymal stem cells support acute myeloid leukaemia cell survival and enhance chemoresistance via contact‐independent mechanism
Source: Br J Haematol. 2024 Nov 10;206(3):858–63. doi: 10.1111/bjh.19884 (PMC11886936; doi:10.1111/bjh.19884)
Supplement: Supplementary file 1 — Data S1. [file BJH-206-858-s001.pdf]

## Supplementary Information

## Supplementary Materials and Methods

### Patient Samples

Bone marrow (BM) and peripheral blood (PB) samples were obtained with written informed consent, in accordance with the declaration of Helsinki and with West of Scotland Research Ethics Committee approval (20-WS-0066), from patients or guardians of paediatric patients with AML in the West of Scotland and from patients taking part in the MyeChild01 clinical trial within the United Kingdom, and from the Bloodwise Childhood Leukaemia Cell Bank (Now VIVO biobank) (MTA 17-009).

### Primary sample preparation

Mononuclear cells (MNC) were isolated from primary AML BM or PB samples transported in EDTA containing blood collection tubes, using density gradient cell separation with Histopaque Density 1077 solution (Sigma-Aldrich), according to manufacturer's instruction. Isolated MNCs were used fresh for the isolation of paediatric MSCs (pMSC) or were cryopreserved and stored in liquid nitrogen (LN) at -185°C.

pMSCs were isolated from cryopreserved or freshly isolated MNCs using the plastic adhesion method. MNC were seeded at 0.5-1.5x$10^6$/ml in plastic ware (media volume 0.32ml/$cm^2$) in MSC culture media (DMEM, 10% MSC growth medium (Promocell), 2mM L-Glutamine 1% penicillin-streptomycin). After 72 hours, the media was changed leaving adherent cells behind in the flask. Media was changed every 2-3 days until cells were 70-90% confluent. pMSCs were passaged using Accutase (Gibco, Thermo-Fisher) and used for experiments between passage 2-5. pMSCs were cryopreserved and stored at LN at -185°C. To ensure that pMSCs met the ISCT minimal criteria for the definition of MSCs, all pMSCs had their phenotype and ability to differentiate confirmed by flow cytometry and in vitro

differentiation assays. pMSC phenotype was carried out using 50,000 cells used per test. Unstained cells and fluorescence minus one (FMO) controls for CD73, CD105 and CD90 were used as controls. eComp beads were used for compensation. A panel of antibodies (CD33, CD11b, CD105, CD90, CD73, CD45, CD14, CD19, HLA-DR) were used as outlined in ISCT criteria using the flow cytometry method.

**Adipogenesis Assay**

MSCs were passaged when cells were 70-90% confluent and TB exclusion cell counts were performed. Cells were seeded at a density of $10^4$ cells/$cm^2$ (i.e 20,000 cells per well of a 24 well plate) in MSC culture media until a confluent layer had formed. Induction media (IMDM, FBS 10%, P/S 1%, Dexamethasone 1µM, Insulin10µg/ml, Indomethacin 50µM, 500µM MIX) was then added to test wells and MSC culture media to the control wells. After 72 hours, the induction media was replaced by maintenance media (IMDM, Insulin 10µg/ml, P/S 1%, FBS 10%) and left for 24 hours. This process was repeated until cells had been in culture for 14-21 days. The experiments were set up in triplicate for cell staining with a control well. For Oil Red O staining to be performed, 10% formalin solution was added to the wells for 30 mins to fix the cells. A stock solution of Oil Red O was made by adding 100mg of Oil Red O powder to 30ml of 99% isopropanol. A working solution was made by a 3:2 dilution of Oil Red O stock solution with distilled water and added to the fixed cells. After 30 mins of staining, the cells were washed with distilled water and visualised under an inverted light microscope and images captured using Cell^B analySIS Image Processing software. Images were edited using Fiji (Image J) software.

**Osteogenesis Assay**

MSCs were seeded into 24 well plates as described above. Osteogenesis media (StemPro) or MSC culture media was added to test and control wells respectively. The media was changed every 2-3 days for 14-21 days. The cells were then fixed with 10% formalin,

washed and stained with Alizarin Red Staining Solution. 250μl of Alizarin Red Staining Solution was added to the wells and incubated for 20 mins. The wells were then washed x4 with distilled water. The wells were then visualised by light microscopy and images captured using Cell^B analySIS Image Processing software. Images were edited using Fiji (Image J) software.

**Chondrogenesis Assay**

MSCs were harvested once 70-80% confluent and TB cell counts performed. A cell suspension of $1.6x10^7$ live cells/ml was made ($1.6x10^6$ cells in 100µl). 5µl droplets of cell suspension was added to the centre of wells in a 96 well plate. The cells were left in an incubator at 37°C for 2 hours, then chondrogenesis media (StemPro) or MSC culture media added to test and control wells. The media was changed every 2-3 days for greater than 14 days. The cells were fixed with 10% formalin solution and stained with Alician Blue Staining Solution. Alician Blue binds proteoglycans that are synthesised by chondrocytes, staining the pellets blue. Following staining for 15 mins, wells are washed with 3% acetic acid. After the final wash, distilled water was added to neutralise the acid and the wells were visualised under light microscopy and images captured using Cell^B analySIS Image.

**Primary pMSC co-culture experiments**

pMSCs were seeded at a density of $10^4/cm^2$ in MSC culture media and left for 72 hours or until >80% confluent. Cryopreserved primary AML MNCs were thawed and resuspended in human culture media (Stemspan SFEM, Stem Cell Technologies), 5% myelocult 1500 (Stem Cell Technologies) supplemented with recombinant human FLT3L, IL-3, IL-6 and stem cell factor (PeproTech). Initial TB exclusion cell counts were performed and MNCs were seeded in equal numbers onto the confluent layer of pMSCs, with or without a transwell, or directly into an empty well for liquid culture. The primary AML MNCs were seeded at a

density of $0.5x10^6$-$1.5x10^6$ live cells/ml. Kasumi 1 AML cells, in the exponential growth phase, were seeded at a density of $0.2x10^6$/ml live cells/ml in RPMI supplemented with 20% FBS. The cells were added to the wells in 500µl of culture media and left for 12-24 hours before the addition of GO (MTA Pfizer 66602-WI188806) or Ara-C (Selleckchem) to the drug treated wells or culture media to the vehicle control (VC) wells to give a final volume of 1ml. For all human samples, the dose of 250ng or 500ng of GO was used. A calculated IC50 dose was used in Kasumi 1 co-culture experiments for GO (150ng/ml) and Ara-C (150ng/ml). The cells were harvested after 48 hours (Ara-C) or 72 hours (GO) post drug treatment and analysed using flow cytometry, including apoptosis assays and cell cycle assays.

**HS5 co-culture experiments**

HS5 cell line were seeded at a density of $10^4$ /cm2 in culture media and left for 72 hours until more than 80% confluent. As above, Kasumi 1 cells were added to a pMSC layer, with or without a transwell, or directly into an empty well for LC at a density of $0.2x10^6$ live cells/ml. The cells were added in 1 ml of culture media and left for 12-24 hours before the addition of Ara-C (150ng/ml) or Ruxolitinib (0.5µM, Selleckchem) to give a final volume of 2 ml. The cells were harvested after 48 hours and analysed using flow cytometry.

**FACS of primary AML cells from CC**

The primary AML samples being used for the RNA sequencing experiment were collected from LC and CC. TB exclusion cell counts were performed, and samples were stained with CD45 and DAPI to exclude non-viable cells. Unstained LC (MNC alone), unstained and stained pMSCs alone, and FMO controls were used to ensure an appropriate gating strategy was used. pMSCs were excluded from the collection based on size and granularity and CD73+ staining (for HS5) and excluding CD45+ staining.

**Apoptosis assay**

Cells were stained with Annexin V and DAPI in 50µl of HBSS (1:100 dilution for each stain). After 15 mins of incubation at RT in the dark, 200µl of HBSS was added to samples. The samples were immediately processed on the BD FACS Canto II. The samples were gated on FSC/SSC and then assessed for viable cells and cells entering early and late apoptosis using Annexin V and DAPI staining. The initial gating strategy for primary sample was based on FSC/SSC, which excludes large pMSC and small lymphocytes and includes the blast containing population. The live population is gated for CD45 expression. To quantify the number of live blasts the number of events in the CD45+ population was divided by the number of events in the initial gate, to give a percentage of the number of live CD45+ blast cells in each sample.

**Cell Cycle Assay**

The BD Cytofix/Cytoperm Fixation and Permeabilization kit (BD Biosciences) was used to prepare cells for cell cycle analysis as per manufacturers instructions, prior to staining with DAPI (1:1000 dilution). Cells were incubated for ten mins at RT in the dark and then samples were immediately run on the BD FACS Canto II. DAPI was acquired on the linear scale. Plots were objectively analysed using the FlowJo cell cycle analysis tool and the Watson (pragmatic) equation to identify the percentage of cells within each stage of the cell cycle.

**RNA extraction**

FACS sorted CD45+ cells were collected and a cell pellet was produced. RNA was then extracted using the Arcturus PicoPure RNA isolation kit according to the manufacturer's protocol. On column DNAse treatment was performed using an RNA-free DNAse kit (Qiagen). RNA quality control was performed to assess RNA quantity, using a Qubit

analyser, and RNA quality by performing RNA electrophoresis to assess RNA integrity using nano bioanalyser chips.

**RNA sequencing**

Primary AML samples from pMSC co-culture experiments with RNA quantity of >100ng and a satisfactory RNA integrity number (RIN) of greater than 5 were selected and RNA sequencing (PolyA selection, Paired Ends, 30 million reads) was performed by Glasgow Polyomics (University of Glasgow). FACS sorted CD45+ AML cells from pMSC CC or LC for 6 different patients were processed. Raw data files in the form of fastq files were assessed for the quality of sequencing using FastQC (V 0.11.9) and sequences were trimmed using fastp (V 0.23.2).

The reference genome (Homo_sapiens.GRCh38.dna_sm.primary_assembly.fa.gz) was obtained via Ensembl (http://ftp.ensembl.org/pub/release-91/fasta/homo_sapiens/dna/, 11/01/2018). Fastq files were aligned to the index genome using STAR (V2.7.10a). Count matrix was generated using FeatureCounts (V2.0.3). PCA plot and identification of differentially expressed genes between the culture conditions was performed using DESeq2 (V.1.37.6), with LC treated as the control group. Log2fold change shrinkage was performed using the apeglm shrinkage method. Heatmaps and volcano plots were made using complexHeatmap (Version 2.13.1) and a modified ggplot function respectively.

Gene set enrichment analysis (GSEA) was performed using differential expression data from DESeq2, ranked by descending log2fold change, using the fGSEA package. Different pathway gene groups e.g. C2_Kegg, C5_GO_BP, and custom gene sets subsetted from all groups after searching for pathways of interest were selected from the Molecular Signatures Database (MSigDB) and loaded into the fGSEA package using MiSigDBR package (version 7.5.1). The fGSEA function was run using a p-value boundary (eps) of 0 and npermsimple of 10,000. The custom gene sets included 60 pathways related to cytokines,

115 pathways involving extracellular matrix adhesion and integrins and 33 pathways relating to IL-6 and VEGF-A. Statistically significant pathways were selected based on an adjusted p-value of <0.05 and a normalised enrichment score (NES) of >1.4.

**Multiplex Immunoassay**

MSC were seeded at $10^4$/$cm^2$ in 48 well plates in a total volume of 500µl of media. AML MNC were seeded at $0.2x10^6$/ml onto a pre-prepared confluent pMSC layer or directly into wells (LC) in 500µl of human culture media without cytokines supplementation. Cell culture supernatant were collected at 24, 48 and 72 hours. The cell suspensions were collected from the wells and centrifuged at high speed (3000g for 10 mins).

A Cytokine/Chemokine/Growth Factor 45-Plex Human ProcartaPlex Panel 1 kit (Thermo-Fisher) and a custom designed 8-Plex ProcartaPlex Panel kit (ThermoFisher) were used for cytokine profiling of the cell culture supernatants, according to manfacturers instructions. The cell culture supernatant was not diluted for use. Samples and standards were performed in duplicate. Frozen cell supernatant samples were thawed on ice and centrifuged at 10,000g for five mins prior to use. Bio-Plex Manager software and ProcartaPlex analyst software was used to analyse the Procartaplex multiplex immunoassay data. The complexHeatmap package (V2.13.1) was used to create a heatmap of the data set scaled to a z-score across the samples for each cytokine.

**Cell Derived Matrices (CDM)**

Plates were prepared by coating with 0.2% gelatin, then crosslinked with 1% glutaraldehyde and left at RT for 30 mins. After washing steps, wells were quenched with 1M glycine solution for 20 mins at RT. After further PBS washes, the wells were incubated with MSC culture media for 30 mins at 37°C/5% $CO_2$. pMSCs were seeded at $1.3x10^4$/$cm^2$ in the prepared plates and left for 72 hours in MSC culture media. After a confluent BMSC

layer had formed (72 hours), media was replaced with MSC culture media supplemented with 50µg/ml ascorbic acid. The ascorbic acid media was then refreshed every 2-3 days for seven days. The media was then aspirated and the plates were washed with PBS containing both Calcium and Magnesium (D-PBS). Extraction buffer (DPBS with MgCl2 and CaCl2, 20mM NH4)H, 0.5% Triton X-100) was added to decellularize the matrix and removed after two mins, and then two D-PBS washes were performed. DNAse I 10µg/ml solution was added, and the plates were incubated at 37°C/5% $CO_2$ for 30 mins. The DNAse I solution was removed and a further two washes with D-PBS was performed. The CDM were then ready to use immediately or were stored in D-PBS with anti-microbial supplementation (1% Pen/Strep) for up to four weeks at 4°C. The CDM were used in co-culture experiments with the Kasumi 1 cell line. The co-culture experiments were set up as described above, with both a pMSC and CDM layer being used within experiments.

**Immunostaining of CDM**

CDM was fixed with 4% paraformaldehyde for 15 mins at RT. Two PBS washes were performed, then the CDM were blocked for one hour at RT in 1% bovine serum albumin (BSA) in PBS and then incubated with the primary antibodies (anti-fibronectin-1 (FN1, BD Biosciences), and anti-collagen 1 (abcam)) for two hours at RT or overnight at 4°C. Following two PBS washes and one D-PBS wash, the CDM were incubated with the secondary antibodies (alexa Fluor 488 and 555) for one hour. The CDM were then washed three times in PBS and imaged using the Nikon Eclipse fluorescent microscope. Fiji (Image J) software was used for image analysis.

**Western blot**

Protein lysates were prepared using RIPA buffer with phosphate and protease inhibitors. Proteins were resolved on SDS-PAGE gel and transferred to nitrocellulose membrane. The membranes were blocked using dried-skimmed milk (5% on TBST) then incubated with

primary antibodies. Primary antibodies were as follows: Phospho-STAT3 (Tyr705),Total STAT3, phospho-ERK, Total ERK, GAPDH (Cell Signalling Technology (UK)). Thereafter, the membranes were incubated with Goat anti-Mouse IgG (H+L) secondary Antibody HRP (ThermoFisher Scientific (UK)) and ECL Rabbit IgG HRP-linked secondary antibody (Amersham (UK)). The proteins were visualized using chemiluminescence substrate (ThermoFisher Scientific (UK)) using Li-COR (ODYSSEY-Fc).

**Statistical Analysis**

Unless otherwise stated data represents mean value +/- standard deviation (SD). For statistical analysis comparing 2 groups a paired or unpaired, two-tailed, Students t-test was used. A paired t-test was used for primary AML samples, when the same sample was compared in two different conditions i.e LC and CC. A p-value of $<0.05$ was considered significant. Graphs and statistical analyses were performed using Microsoft Excel and Graph Pad Prism version 9.

A

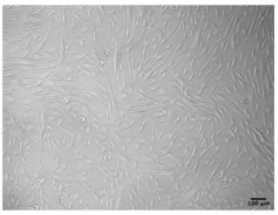

B

| ISCT Minimal Experimental Criteria for MSC 2006 |
|---|
| Adherence to plastic. |
| ≥95% of the MSC population must express CD73, CD90 and CD105 as measured by flow cytometry. ≤2% of the MSC population must express CD34, CD45, CD11b or CD14, CD79 or CD19, and HLA-DR. |
| In vitro differentiation: osteoblasts, adipocytes and chondroblasts. |

C

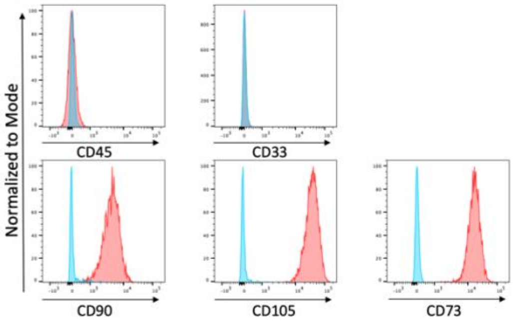


D

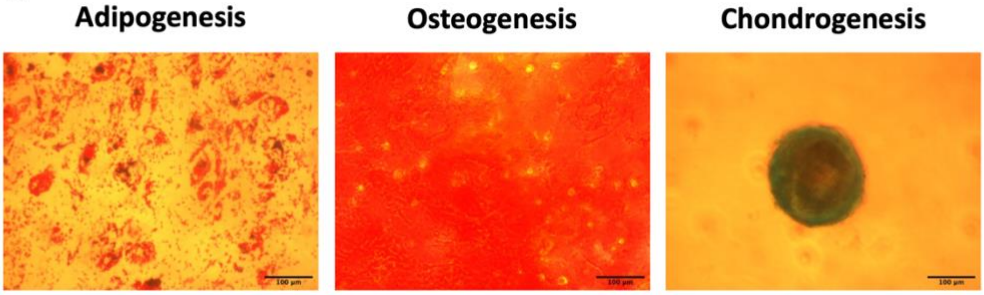


E

| Patient Sample ID | Experiment | Age (yrs | Sex | Source | Cytogenetics/Molecular abnormalities |
|---|---|---|---|---|---|
| AML035 | BMSC isolation | 44 | Male | Bone marrow | CEBPA |
| AML037 | BMSC isolation | 19 | Male | Bone marrow | +8, del(9), FLT3-ITD |
| AML063 | BMSC isolation | 49 | Male | Bone marrow | Normal karyotype |
| FR0268 | RNA sequencing | 2.83 | - | Bone marrow | FLT3-ITD |
| FR0551 | RNA sequencing | 9.3 | Female | Bone marrow | Core binding factor AML |
| FR0736 | RNA sequencing | 3.9 | - | Bone marrow | MLL rearranged |
| FR0797 | RNA sequencing | 3 | Male | Bone marrow | MLL rearranged t(6;11) |
| PAML001 | BMSC isolation, CC experiments, RNA sequencing, qPCR | 1.3 | Female | Bone marrow | NUP98-NSD1 rearrangement |
| PAML002 | BMSC isolation, CC experiments, RNA sequencing, qPCR | 5 | Male | Bone marrow | del (9) |
| PAML003 | CC experiments, | 14 | Male | Bone marrow | GATA2 mutation |
| PAML015 | BMSC isolation | 15 | Male | Bone marrow | t(11;19) |
| PAML019 | CC experiments, CFC assay | 4 | Male | Bone marrow | t(9;17) |
| PAML022 | BMSC isolation | 3.5 | Female | Bone marrow | Inv(16) |
| PAML026 | BMSC Isolation | 0.3 | Female | Bone marrow | t(11q23) |
| PAML028 | BMSC isolation, CC experiments, qPCR, CFC assay | 3.5 | Male | Bone marrow | t(10;11) |
| PAML029 | CC experiments, CFC assay | 13 | Female | Bone marrow | Normal Karyotype, NPM1 mutations |
| PAML030 | BMSC isolation | 3 | Female | Bone marrow and peripheral blood | Complex karyoptype |
| PAML031 | BMSC isolation | 3 | Female | Bone marrow | t(10;11) |
| PAML033 | CC experiments | 13 | Male | Bone marrow | t(11;17) |
| PAML034 | BMSC isolation, CC experiments, | 9 | Female | Bone marrow | Normal karyotype, FLT3 and NPM1 mutation |
| PAML035 | CC experiments | 17 | Female | Bone marrow | Unknown |

Figure S1. A) Representative microscopic image showing AML pMSCs (n=9) during culture (passage 3). Scale bar represents 100µm. B) Table displaying the minimal experimental criteria for the definition of MSC as defined by the ISCT. C) AML pMSCs were authenticated by flow cytometry for the CD45-CD90+CD105+CD73+ phenotype using flow cytometry. Representative histograms are shown here (n=9). The unstained pMSC or FMO control is represented by the blue histogram and the stained BMSC by the red histogram. D) Representative microscopic images of in vitro differentiation assays of AML pMSCs (n=9). The image in the left panel shows adipocytes (Red Oil O stain), the middle panel osteoblasts (Alizarin red stain) and right panel chondrocytes (Alician blue stain). Scale bars represent 100µm. E) Table showing primary human pAML samples patients characteristics.

Figure S1

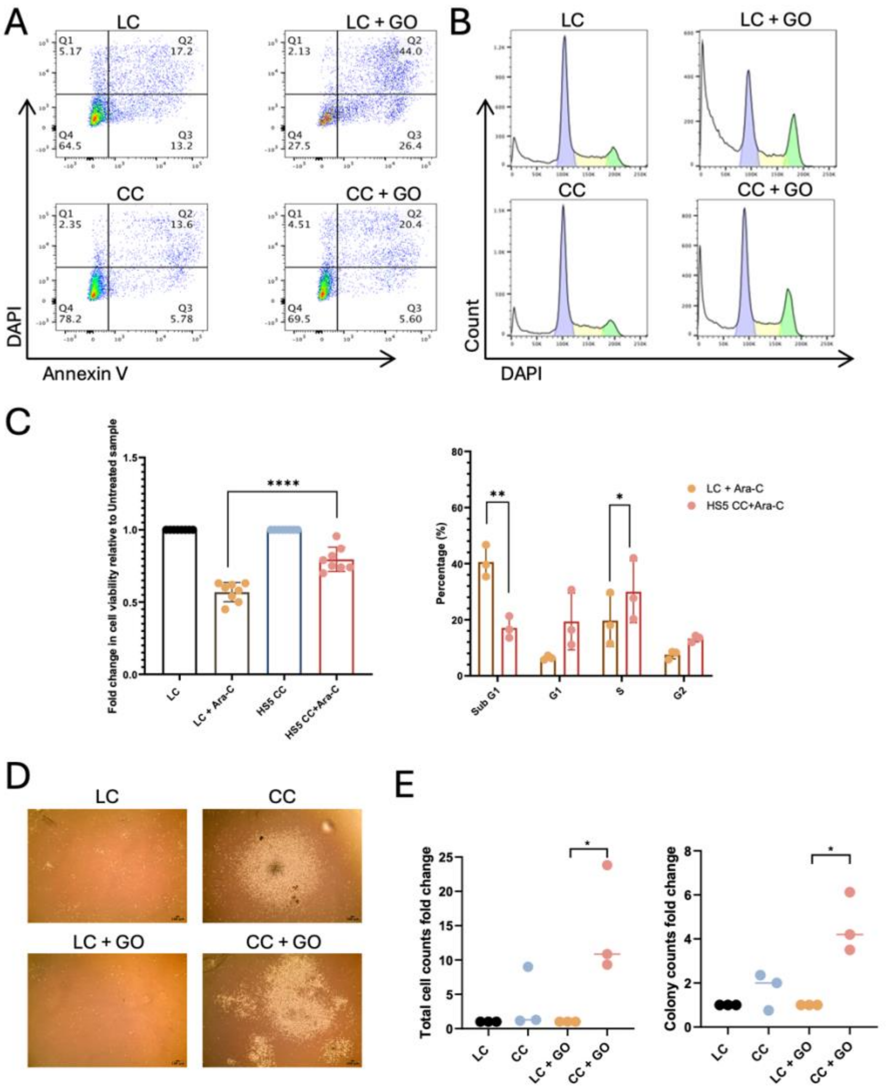


Figure S2. A) Representative flow cytometry plots of an apoptosis assay of Kasumi-1 pAML cells in CC with primary pMSCs compared to LC (n=10 biological replicates). B) Representative flow cytometry plots of cell cycle using DAPI staining (n=9 biological replicates). Sub GI = unfilled, G1 = purple, S = yellow, G2 = green. C) Graphs show Fold change in the percentage of live (Annexin V-/DAPI-) Kasumi-1 AML cells from an apoptosis assay (left panel) and percentage of cells in each phase of the cell cycle (right panel), in LC and HS5 CC after Ara-C drug treatment compared to the respective untreated sample. n=3 biological replicates. D) Representative microscopic photographs of a colony forming assay from a patient sample of FACS sorted CD45+ cells from LC or CC with and without GO treatment (n=3). E) Total cell counts (left panel) and total colony counts (right panel) for each condition after 14 days in colony-forming assay normalised to respective LC to allow for sample variation (n=3). Graphs show mean and P-values were determined using a two-sided unpaired Student's t-test (* p<0.05, **p<0.01, **** p<0.0001).

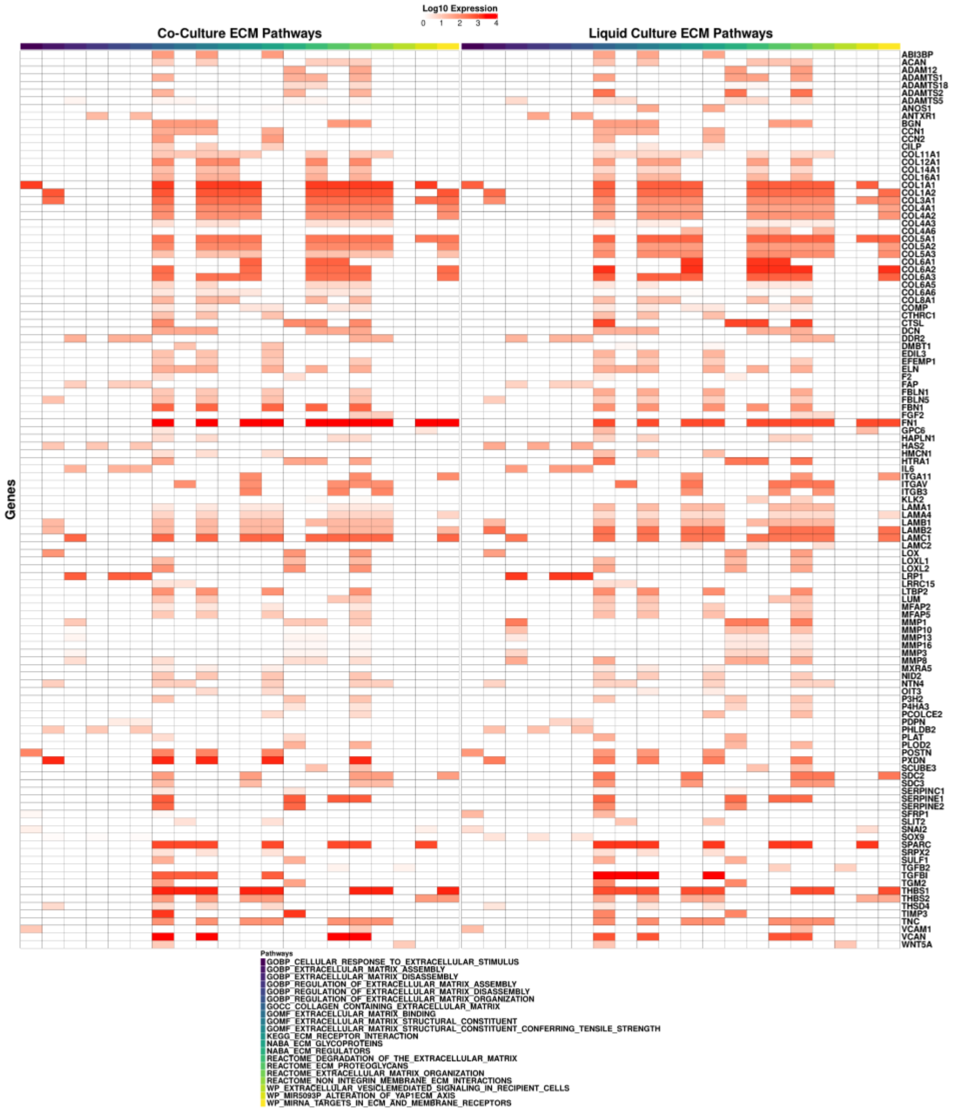


Figure S3. A) Heat map comparing average log10 expression levels between LC and CC. Shown are the genes present in the leading edge of the pathways related to the extra cellular matrix from the gsea analysis

Figure S3

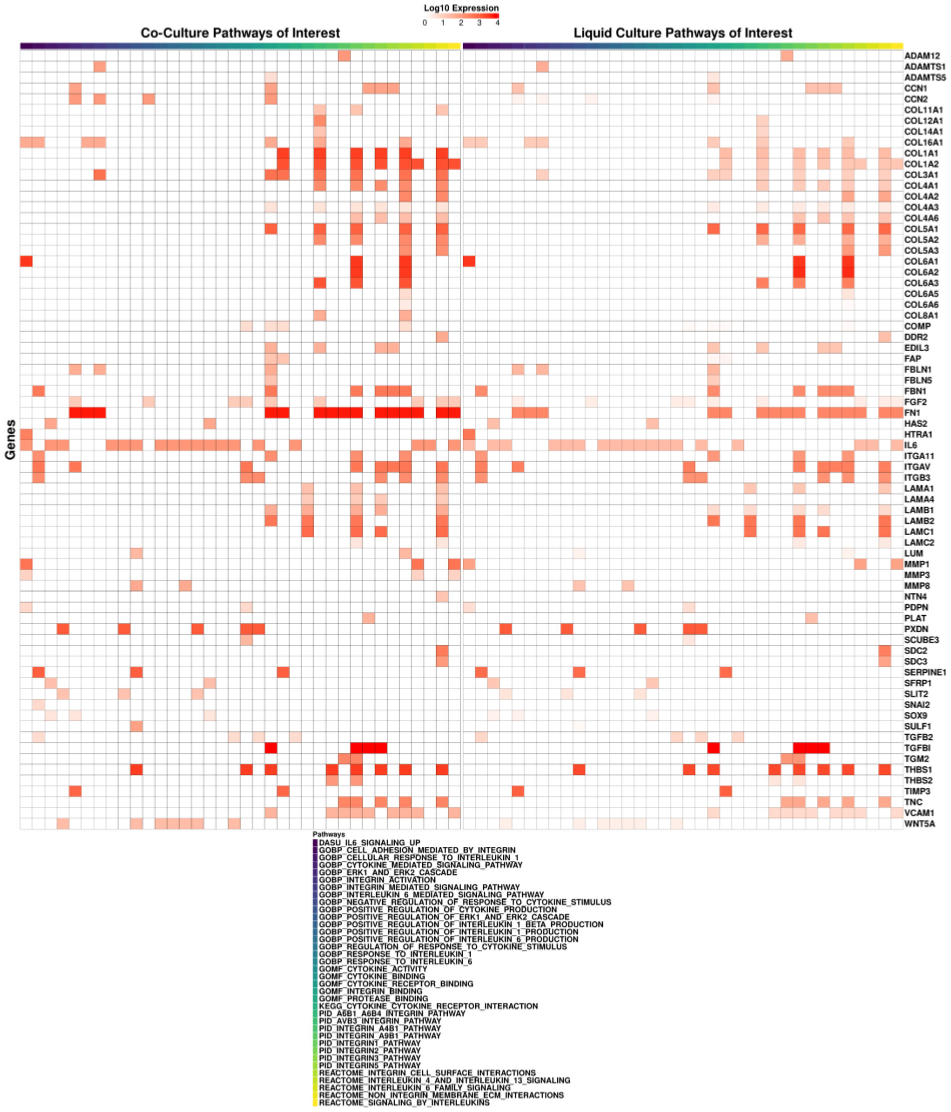


Figure S3. B) Heat map comparing average log10 expression levels between LC and CC. Shown are the genes present in the leading edge of the pathways related to integrins, cytokines, interleukins and ERK signaling.

Figure S3

A

| pAML sample | pMSC |
|---|---|
| PAML001 | PAML001 |
| PAML002 | PAML002 |
| PAML003 | PAML015 |
| PAML019 | PAML031 |
| PAML027 | PAML034 |
| PAML028 | PAML026 |
| PAML033 | PAML026 |
| PAML034 | PAML034 |
| PAML035 | PAML026 |

B

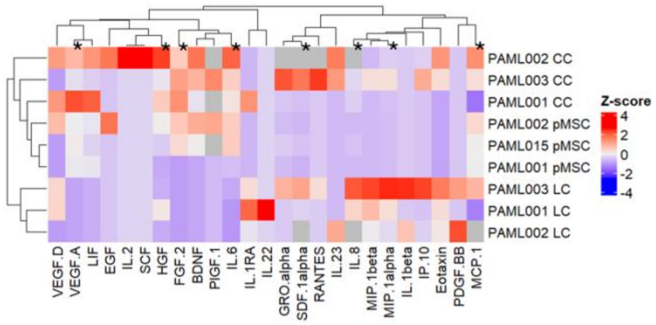


C

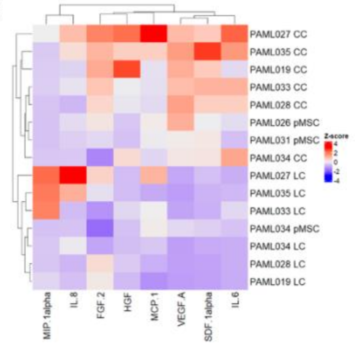


D

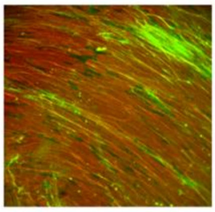


Figure S4. A) pAML and pMSC sample pairs for cell culture supernatant collection. B) Hierarchical clustering analysis heat map from a 45-plex immunoassay displaying cytokines with detectable levels. Each row shows the z-score data for each P-AML sample in LC or CC or pMSCs alone (n=3) at 72 hours. Each column shows the data for a specific cytokine. Red indicates a positive z-score and blue a negative z-score. The z-score is the number of standard deviations the value (e.g. concentration pg/ml) is above or below the mean value. The grey boxes represent values that were excluded from analysis due to a coefficient of variation (CV) of the replicates being greater than 30%. * indicates cytokines present on both the 45- and 8-plex immunoassay. (C) Hierarchical clustering analysis heat map showing z-scores for 8 cytokines (columns) for each P-AML sample in the different culture conditions (rows) at 72 hours: LC (n=6), CC (n=6), pMSCs alone (n=3). D) A representative fluorescent microscopy image of the cell derived matrix (CDM) confirming the presence of FN1 (red) and COL1A1 (green) (n=2 biological replicates)

Figure S4
